# Research on the impact of different teaching methods on students’ spatial ability and three-dimensional geometric thinking

**DOI:** 10.3389/fpsyg.2026.1744734

**Published:** 2026-02-12

**Authors:** Tanjun Ma, Wenlan Zhang

**Affiliations:** 1 Faculty of Teacher Development, Shaanxi Normal University, Xi’an, China; 2 Faculty of Education, Shaanxi Normal University, Xi’an, China

**Keywords:** gesture, spatial capability, student, three-dimensional geometric thinking, visualization technology

## Abstract

Spatial ability is a fundamental cognitive skill that is a strong predictor of success in STEM education. Three-dimensional geometric thinking is the formalized expression of spatial ability when solving three-dimensional geometric problems. Although technology and embodied cognition are regarded as potential means to promote students’ spatial ability and three-dimensional geometric thinking development, currently there are few studies focusing on the relative influence of different teaching methods on students’ spatial ability and three-dimensional geometric thinking, as well as the moderating effect of students’ initial spatial ability on teaching methods. Based on the theory of multimedia learning and cognitive load, this study designs a teaching method that integrates GeoGebra (a dynamic visualization tool) and gestures (a form of embodied cognition). The study adopted a quasi-experimental pre-test–post-test control group design. The differences in the independent and integrated effects of GeoGebra and gesture cognitive enhancement methods, as well as the moderating role of students’ initial spatial ability, were explored. The research results show that: (1) Although the spatial ability scores of all teaching groups have significantly improved from the pre-test to the post-test, there is no significant difference among the three teaching methods. (2) There are significant differences in the influence of the three teaching methods on students’ three-dimensional geometric thinking. (3) Students’ initial spatial ability does not significantly moderate the effect of teaching methods, but it is the main predictive factor for the post-test of spatial ability and three-dimensional geometric thinking. (4) The overall influence of students’ initial spatial ability on three-dimensional geometric thinking is greater than that of teaching methods. This study provides reference suggestions for teachers to design spatial ability and three-dimensional geometric thinking cultivation programs that match the differences in students’ spatial abilities.

## 1 Introduction

Spatial ability (SA) and three-dimensional geometric thinking (3D-GT) are the core elements of three-dimensional geometry learning. SA is the cognitive basis of 3D-GT (Pittalis and Christou, 2010), and 3D-GT is the mathematical and formal expression of SA. Studies have shown that there is a strong correlation between students’ SA and their mathematical learning achievements (Mix and Cheng, 2012), and early SA can predict subsequent achievements in mathematics and science fields (Wai et al., 2009). Although there are significant differences in SA among groups of different genders and ages (Geiser et al., 2008), However, SA has a certain degree of plasticity and can be improved to a certain extent through methods such as video game training, spatial course learning and spatial task training (Uttal et al., 2013). Therefore, paying attention to the cultivation of students’ SA and 3D-GT in three-dimensional geometry teaching is of great value and significance for their geometric learning and future mathematical achievements (Gutierrez and Boero, 2006). Three-dimensional geometry contains a large number of spatial elements and three-dimensional geometric reasoning. Students may encounter learning difficulties in the process of spatial visualization (Alghadari et al., 2020) and geometric reasoning (Pittalis and Christou, 2010). Therefore, the difficulty in three-dimensional geometry teaching lies in helping students lacking SA to overcome spatial cognitive barriers and assisting those with higher SA to develop geometric thinking. The adoption of three-dimensional geometry teaching methods by teachers that match students’ initial SA has become the key to breaking through the key points and difficulties in three-dimensional geometry learning.

GeoGebra software is a typical dynamic visualization software applicable to the learning of various modules such as geometry, algebra, data, drawing, statistics and calculus (Keong et al., 2005). A large number of studies have explored the role that GeoGebra plays in geometric learning and cognition. The relevant results show that students who used GeoGebra software for geometric concept learning achieved higher grades than those who used traditional methods (Eyyam and Yaratan, 2014; Moses et al., 2012). GeoGebra software helps students conduct visual and understandable exploration of geometric content, thereby improving students’ attitudes toward geometry (Arbain and Shukor, 2015; Mathevula and Uwizeyimana, 2014; Niyukuri et al., 2020; Ocal, 2017). Virtual interaction through video games (Spence and Feng, 2010), geometric dynamic software drawing (Kepceoglu, 2018), and augmented reality (AR) (Supli and Yan, 2024) has a positive impact on the improvement of SA and drawing skills. Other studies have shown that the use of GeoGebra in spatial geometry courses can have a significant positive impact on students’ spatial mathematical abilities (Izzati et al., 2024) and spatial visualization abilities (Suparman et al., 2024; Izzati and Farizi, 2025). The use of GeoGebra in the process of geometric geography learning can also improve students’ spatial visualization abilities (Prasetya et al., 2025). However, studies have shown that students with low SA become cognitive overloaded due to the existence of dynamic visualization models, while students with high SA benefit from it because their total cognitive load remains within the range of working memory (Huk, 2006). Another study suggests that compared with learners with high SA, the performance of learners with low SA is more positively influenced by the desktop virtual technology (VR) learning environment (Lee and Wong, 2014) and 3D virtual world simulation (Merchant et al., 2013). It can be seen from this that dynamic visualization technologies such as GeoGebra are, to a certain extent, conducive to students breaking through spatial cognitive barriers, improving their geometric learning performance and attitude, and developing their SA and 3D-GT. However, the impact of dynamic visualization technologies such as GeoGebra on the learning of students with different initial SA is not always beneficial, and there is no consensus on their learning effects.

Gestures are spontaneous hand movements that accompany speech and are semantically and temporally related to speech (Aldugom et al., 2020). The types of gestures mainly include descriptive gestures (Yang et al., 2023), abstract gestures (Chu and Kita, 2008), indicative gestures and rhythmic gestures (Hostetter, 2008). A large number of studies have explored the role of actions or gestures in geometric learning (Valenzeno et al., 2003) and spatial cognition (Alibali, 2005). Existing studies have shown that gestures are one of the most reliable indicators of visual thinking in mathematics teaching and learning (Presmeg, 1985). Gestures can convey complex three-dimensional spatial relationships and enhance students’ ability to reason about three-dimensional spatial relationship diagrams (Atit et al., 2015). The gesture-based instruction can improve children’s misconceptions about spatial units (Congdon and Levine, 2023). Gestures can not only improve the completion efficiency of spatial visualization problem tasks (Chu and Kita, 2011), but also interact with visual–spatial thinking patterns (Brooks et al., 2018), and can have an impact on thinking and learning (Goldin-Meadow, 2003). People will spontaneously use different gestures when performing mental rotation tasks. The transformation from the initial imitative gestures to abstract gestures reflects the internalization of movement strategies (Chu and Kita, 2008). There are also studies showing that gestures are more effective than direct manipulation of objects in promoting the transfer of mathematical knowledge (Novack et al., 2014). Gestures have a positive impact on the learning of chemical stereoisomers (Ping et al., 2022). Aldugom et al. (2020) suggested that when gestures are present, there is a positive correlation between visuospatial working memory capacity and mathematical learning. Gestures can promote children’s learning of mathematics when actually applied in the classroom (Seccia and Goldin-Meadow, 2024). The main reason is that gestures can reflect mental images, which usually contain spatial information, and these images are the basis for the generation of gestures (McNeill, 1992). Brucker et al. (2022) studied and believed that scholars with higher visuospatial ability could promote learning by observing non-corresponding gestures, even better than the learning effect of observing corresponding gestures, while scholars with lower visuospatial ability had an adverse effect on learning by observing non-corresponding gestures. Yang et al. (2023) suggested that imitating teachers’ gestures in video learning can effectively improve the immediate transfer performance of learners with low SA, but has no effect on learners with high SA. Evidently, gestures play an important role in classroom teaching (Church et al., 2025). Gestures can, to a certain extent, help students overcome obstacles in geometric learning, develop SA and geometric thinking. However, the influence of gestures on the learning of students with different initial SA is not always beneficial, and there is no consensus on its learning effect. Some studies suggest that surface initial SA may moderate the influence of gesture teaching strategies on learning outcomes (Yang et al., 2023).

Based on the existing literature, it can be found that researchers mainly study the teaching methods of 3D geometry from two directions: One is to utilize the dynamic demonstration advantage of dynamic visualization technology to promote the learning and understanding of spatial cognition and geometric concepts (Eyyam and Yaratan, 2014; Moses et al., 2012; Arbain and Shukor, 2015; Mathevula and Uwizeyimana, 2014; Niyukuri et al., 2020; Ocal, 2017). Second, embodied operations such as gestures are adopted to promote students’ expression and reasoning of spatial relationships (Atit et al., 2015), and improve the completion efficiency of spatial problems (Chu and Kita, 2011). Both of these approaches have been proven to have a positive impact on students’ geometric learning outcomes, SA and geometric thinking to a certain extent. However, there is no consensus on the influence of the two teaching methods on the learning outcomes of students with different initial SA. Although the development of SA and 3D-GT is an important manifestation of the learning effect of three-dimensional geometry. However, few studies have focused on the differences in the impact of the two 3-dimensional geometry teaching methods on students’ SA and the development of 3D-GT. There are relatively few studies on the moderating effects of students with different initial SA. From the perspective of personalized teaching, teachers’ selection of matching teaching methods based on students’ differences is the key to promoting students’ development (Tomlinson, 1999). From the perspective of teaching methods, although dynamic visualization techniques such as GeoGebra can effectively present macroscopic and complex graphics, their effects are highly susceptible to individual differences (Hegarty and Waller, 2004; Huk, 2006), and it is difficult to flexibly represent their local spatial relationships. Although gestures can interact with spatial thinking patterns (Brooks et al., 2018), they can visualize microstructures (Atit et al., 2015). However, there is a limitation of insufficient accuracy when characterizing multi-dimensional complex spatial models (Stieff et al., 2016). It can be seen that these two teaching methods are functionally complementary. A single teaching method cannot meet the learning needs of students with different initial SA, and there may be differences in the development of students’ SA and 3D-GT. From the perspective of cognitive load theory, individuals with low SA may experience cognitive load overload when facing complex dynamic visualization models (Huk, 2006) and non-corresponding gestures (Brucker et al., 2022). However, when students with low SA encounter spatial cognitive impairment, cognitive load can be reduced through visualization models (Lee and Wong, 2014; Merchant et al., 2013) and imitating teacher gestures (Yang et al., 2023). It is evident that a single teaching approach cannot effectively manage the cognitive load of students with different initial SA. These two teaching methods are functionally complementary in managing students’ cognitive load. However, most of the existing studies are independent effect tests of dynamic visualization technology and gesture teaching methods, lacking empirical research that integrates the advantages of both and examines whether they can produce a “1 + 1 > 2” gain effect. Given that the comparison of the effects of different teaching methods and the research on the moderating effects of students’ initial SA have significant value and significance for promoting personalized teaching of three-dimensional geometry content. This study uses the typical dynamic visualization software GeoGebra in mathematics teaching as a supporting tool, aiming to answer the following two questions.

Question 1: In the process of learning three-dimensional geometry in high school mathematics, are there significant differences in the impact effects of the GeoGebra teaching method, the gesture teaching method, and the teaching method that integrates GeoGebra and gestures on students’ SA and 3D-GT?

Hypothesis 1: There are significant differences in the impact on students' SA between the GeoGebra teaching method and the gesture teaching method, as well as the teaching method that integrates GeoGebra and gestures.

Hypothesis 2: There are significant differences in the influence of GeoGebra teaching method and gesture teaching method, as well as the teaching method that integrates GeoGebra and gestures, on students' 3D-GT.

Question 2: Does the initial SA of students moderate the influence of different teaching methods on the development of SA and 3D-GT?

Hypothesis 3: Students' initial SA moderates the impact of different teaching methods on SA.

Hypothesis 4: Students' initial SA moderates the impact of different teaching methods on 3D-GT.

## 2 Concept definition

### 2.1 Spatial capability

SA refers to a person’s spatial perception ability and is the general manifestation of intelligence in the spatial cognitive system (Ju and You, 2013). At present, there is no complete unification in the dimension division of spatial capabilities. Lohman (1988) proposed on the basis of literature review that spatial capabilities mainly consist of three dimensions: spatial visualization, spatial orientation and spatial relationship. Linn and Petersen (1985) clearly defined SA as three dimensions: mental rotation, spatial visualization, and spatial perception. Kozhevnikov and Hegarty (2001) hold that spatial capability should also include spatial positioning capability. Among them, spatial visualization is the ability to understand imagined motion in three-dimensional space (French, 1951). Mental rotation is the ability to psychologically manipulate, rotate, distort or reverse an object (Hegarty and Waller, 2005). Spatial orientation is the ability for an individual’s self-reference framework to change relative to the environment, while the relationship between the object reference framework and the environmental reference framework remains unchanged (Hegarty and Waller, 2004). In light of the content characteristics of high school 3D geometry textbooks, this study takes mental rotation, spatial visualization and spatial orientation dimensions as the main dimensions of SA in this research.

### 2.2 Three-dimensional geometric thinking

Geometric thinking encompasses visualization, construction and reasoning processes, and has the function of visual representation of geometric statements (Duval, 1998). Its characteristic lies in the dialectical relationship between geometric images and geometric concepts (Fischbein, 1993). Pittalis and Christou (2010) divided 3D-GT into four parts: the representation of three-dimensional objects, spatial structure, and the conceptualization and measurement of mathematical properties. Van Hiele divides the development of students’ plane geometry thinking into five levels, namely visual, descriptive or analytic, abstract or relation, formal deduction rigor (Clements and Battista, 1992). The geometric content in high school mathematics textbooks mainly includes the recognition of the structural features of basic spatial geometric bodies, the understanding of intuitive diagrams of spatial figures, the calculation of the surface area and volume of simple geometric bodies, the positional relationship between points, lines and surfaces, and the parallelism and perpendicularity between spatial straight lines and planes (Curriculum and Textbook Research Institute, 2019). the curriculum standards require students to be able to learn the main research methods of three-dimensional geometry such as intuitive perception, operational confirmation, reasoning and demonstration, and metric calculation (Ministry of Education of the People's Republic of China, 2020). It can be seen that the geometry learned by students mainly includes three different and interrelated cognitive processes, namely visualization, construction and reasoning (Sudirman et al., 2023). Combined with the content and requirements of three-dimensional geometry, this study takes the theoretical framework of Van Hiele’s plane geometry thinking as the basis for the division of the development of 3D-GT.

### 2.3 Teaching method integrating GeoGebra with gestures

The teaching method that integrates GeoGebra with gestures is to complement the advantages of GeoGebra and gestures. Visualize the microstructure through gestures to promote the “externalization” of internal mathematical thinking. By using GeoGebra to “visualize” complex and abstract mathematical objects, the two are deeply integrated rather than simply superimposed. The modal effect and human movement effect in the cognitive load theory provide a feasible basis for the integration of GeoGebra and gestures. The modal effect assumes that working memory can be divided into multiple independent processors, and using multiple working memories simultaneously can increase the effective working memory capacity (Baddeley, 1992). The human movement effect indicates that when the learning focus is related to human movement, it may not impose an excessive burden on working memory resources (Paas and Sweller, 2012). The human motor system has the ability of mirroring and can be activated by observing the motor movements of others (Rizzolatti and Craighero, 2004). In the teaching method that integrates GeoGebra with gestures, complex dynamic visualization images can be processed through visual working memory. Gestures are encoded in a unique visual–spatial representation format (Paas and Sweller, 2012), which is conducive to increasing the effective working memory capacity. The distraction effect of the cognitive load theory indicates that when two complementary information sources are independently presented to scholars, the psychological integration of scholars will bring a higher cognitive load (Sweller et al., 2019). This provides guidance for the spatial and temporal organization of the integration of GeoGebra and gestures, avoiding the independent presentation of complementary information in space and time. The variation effect of the cognitive load theory indicates that variability increases cognitive load, but it has a positive impact on learning and transfer effects in the case of low cognitive load (Sweller et al., 2019). This provides value and significance for the correct and incorrect descriptions of gestures. Teachers’ description of incorrect spatial relationships through gestures is also helpful for students’ learning.

## 3 Methods

### 3.1 Experimental design

**Figure 1:**
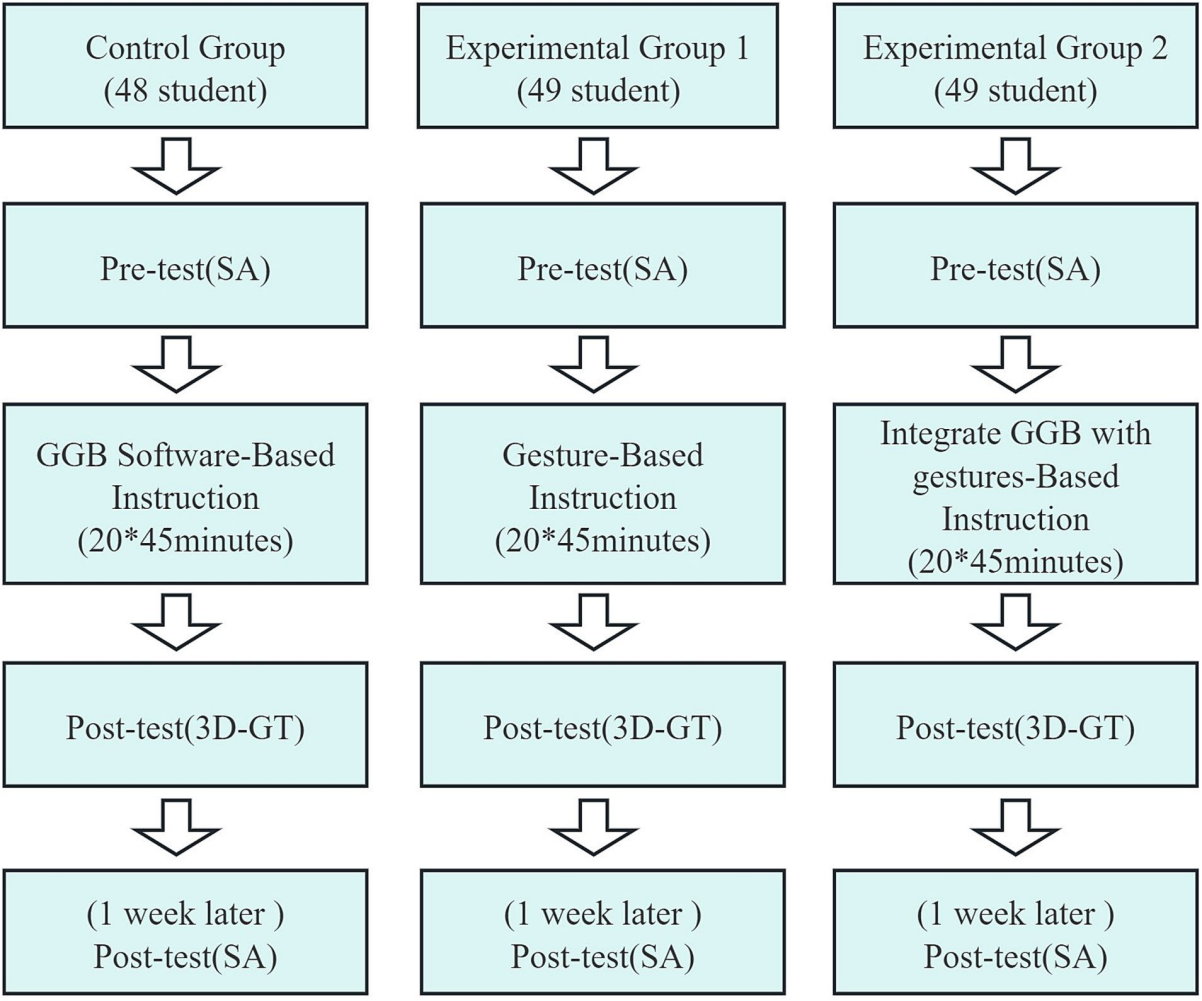
The experimental design of the research GeoGebra (GGB).

This study employed a quasi-experimental pre- and post-test controlled design using intact classes. Based on mathematics test scores and geometry-related assessment results from 10 classes at the same grade level in the participating school, three classes showing no significant differences in overall mathematics performance or geometry-related outcomes were selected as the experimental sample. Due to administrative constraints imposed by the school, random assignment of individual students to different experimental conditions was not feasible. Consequently, the three intact classes were assigned to different instructional approaches. The Control group used the GeoGebra teaching method, the Experimental group 1 used the gesture teaching method, and the experimental group 2 used the teaching method that combined GeoGebra and gestures. Mathematical tests and pre-tests of SA were conducted before the implementation of the experiment. The experimental intervention period was 10 weeks. Two new classes each week adopted the teaching method of experimental design, each lasting 45 min, totaling 20 class periods and 900 min. The teaching content includes all the contents of three-dimensional geometry in mathematics textbooks. Finally, the study carried out Post-test of 3D-GT and SA. For the specific experimental design of the research (see Figure 1).

### 3.2 Participants

All the participants were first-year students from a high school in a provincial capital city of China. The study analyzed the scores of the opening mathematics test of 515 students from 10 classes of this grade using SPSS 25 software. Three classes were selected from them as the experimental subjects. When there were no significant differences in the scores of the mathematics test and the geometry-related items among the three classes, *F*(2,151) = 0.346 and *p* = 0.708 > 0.05. Three classes are taught mathematics by two teachers. The two teachers have the same working history, age, educational background and professional title, and all have a basic understanding of using GeoGebra software. One week prior to the start of the intervention, the two participating teachers underwent a standardized one-week training session conducted by the researchers. The training covered the following: the kinematic standards, timing and semantic interpretation of the geometric gestures used in this study; the principles for developing GeoGebra software courseware; and specific instructional examples demonstrating the integrated use of gestures and GeoGebra. All intervention lessons were delivered using lesson plans co-developed by the researchers and the teachers, which explicitly specified the teaching segments for employing particular gestures and the corresponding trigger points for GeoGebra animations. To monitor implementation consistency, the researchers observed six randomly selected lessons to verify adherence to the intervention protocol. The observation results indicated that both teachers’ use of gestures and execution of key steps were consistent with the design of the lesson plans. A total of 154 students from three classes initially participated in the experiment. Due to the fact that some students were on leave, they were unable to take part in the entire test process, resulting in the absence of a small number of experimental subjects. Eventually, a total of 146 students (75 males and 71 females) effectively participated. The study only conducted statistical analysis on students who effectively participated in the experiment.

### 3.3 The mode of integrating GeoGebra with gestures

In this study, the operation framework integrating GeoGebra and gestures is defined as the “visual presentation–embodied interpretation” collaborative model. The core of this model lies in the teacher’s collaborative use of these two methods. GeoGebra is used to provide an overall and dynamic presentation of geometric objects to build students’ spatial perception. Well-designed gestures are employed for embodied interpretation, aiming to deconstruct the core geometric relationships, key problems, and reasoning logic embedded in the visualized content and make them explicit.

In actual classroom practice, the two modalities are coordinated according to the principle of cognitive synchrony. Most critical instructional moments occur synchronously (e.g., gestures interpreting the geometric relationships in GeoGebra’s dynamic demonstration in real time), while at certain moments they function asynchronously and complementarily (e.g., first using GeoGebra to demonstrate the overall process, followed by gestures to reinforce stepwise understanding).

Throughout this process, the teacher assumes the key role of both cognitive coordinator and interpreter. This role is realized through three primary functions: (1) Cognitive Focus. Guiding students’ attention between GeoGebra visualizations and gestural demonstrations, focusing on key problems. (2) Externalization of Cognitive Processes. Transforming geometric reasoning and logical processes into observable, coherent sequences of gestures actions. (3) Facilitation of Multimodal Integration. Binding visual information (GeoGebra), kinesthetic information (gestures), and semantic meaning (mathematical concepts) through synchronized verbal explanations, thereby supporting the construction of a unified mental representation.

### 3.4 Teaching materials

**Figure 2:**
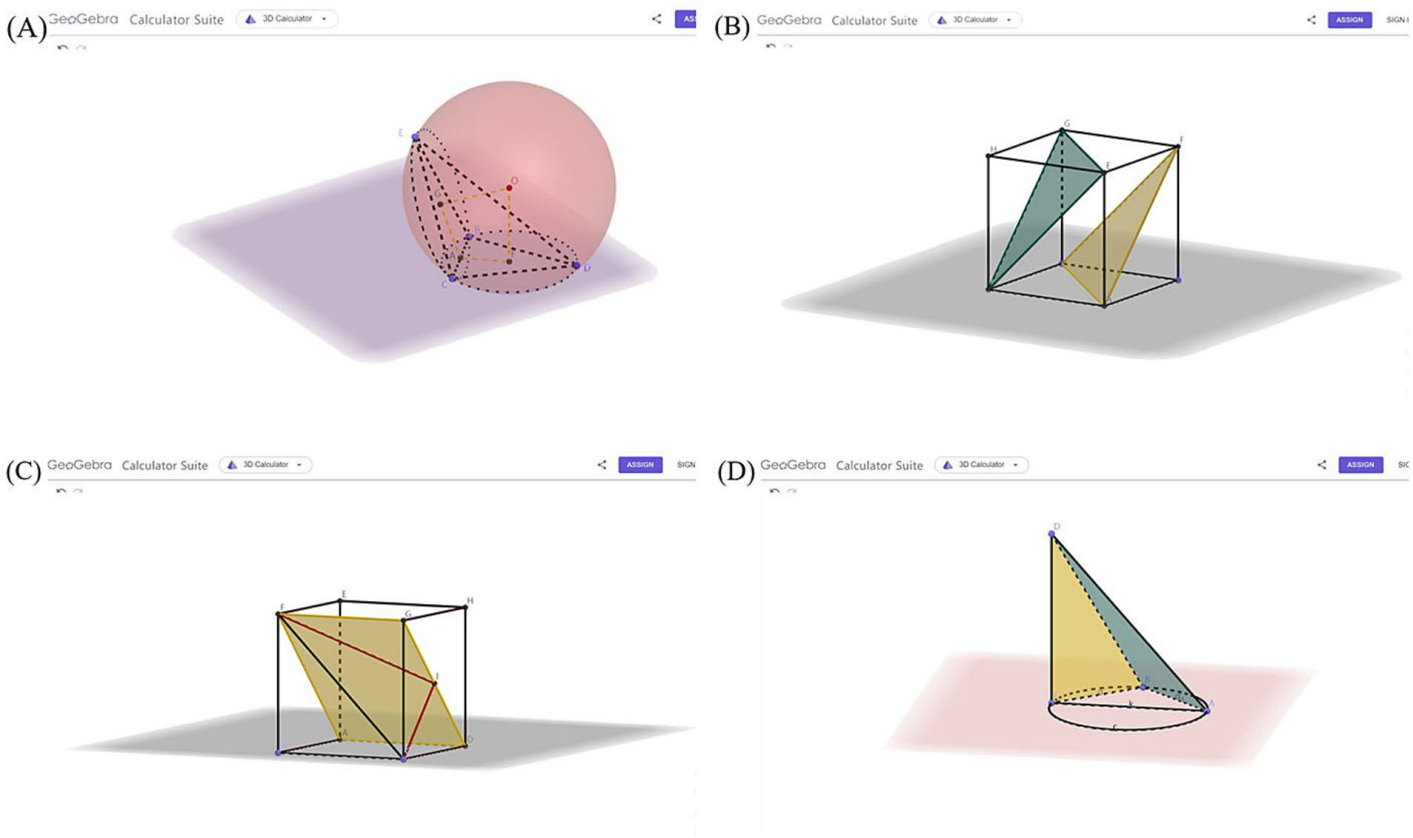
Teaching courseware designed based on the GeoGebra software. Part **(A)** explores the problem of the circumscribing sphere of a triangular pyramid. Part **(B)** explores the proof of the parallel relationship between planes. Part **(C)** is used to investigate the angle between a straight line and a plane. Part **(D)** is used to prove the perpendicular relationship between a line and a plane.

**Figure 3:**
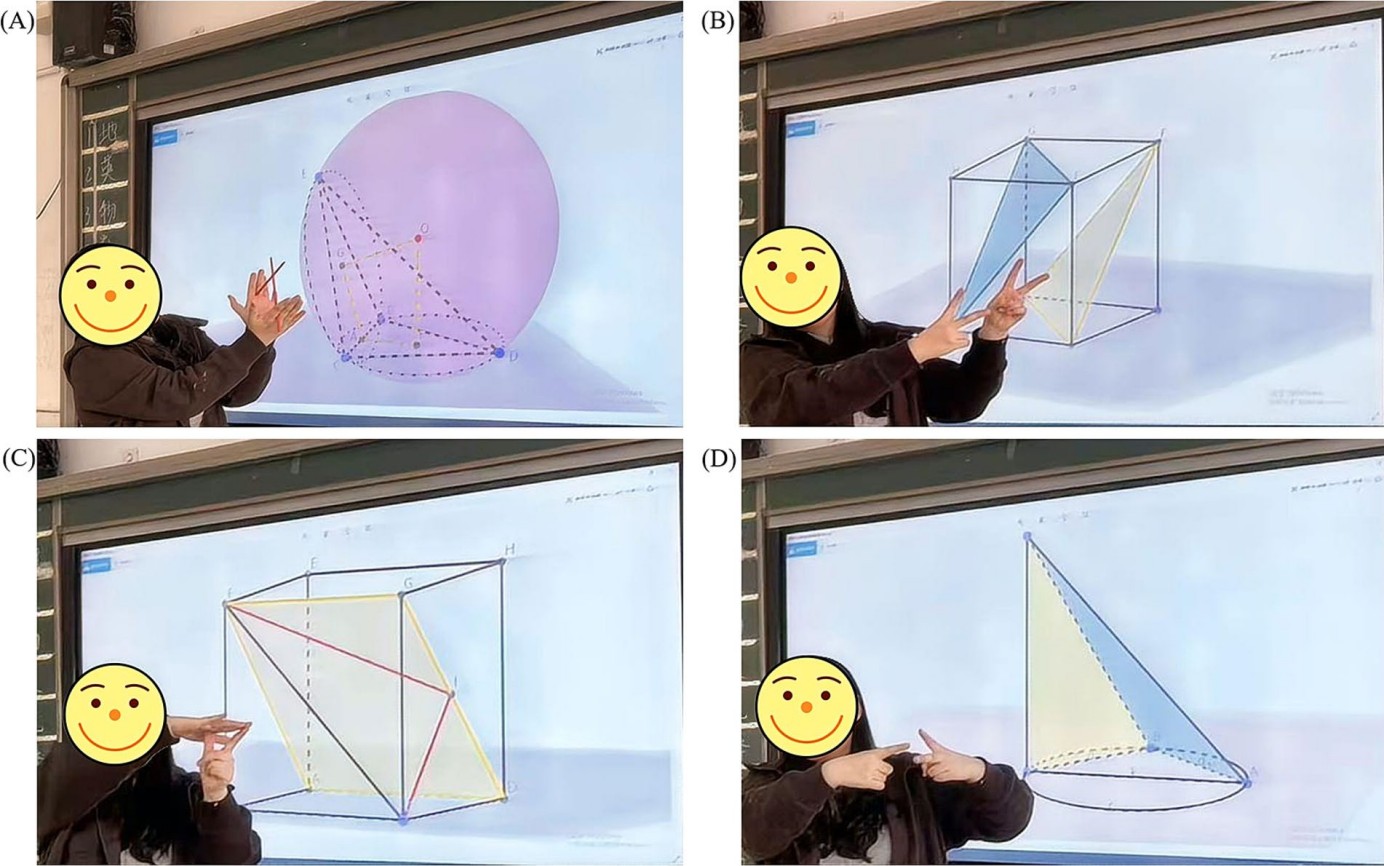
The teaching method integrating GeoGebra with gestures. Part **(A)** discusses the problem of the circumsphere of a triangular pyramid. Gestures supplement and emphasize the method for determining the position of the center of the circumsphere of the triangular pyramid. Part **(B)** explores the proof of the parallel relationship between planes. The gestures further supplemented and strengthened the proof of the parallel relationship between two planes by using two sets of two intersecting and mutually parallel lines. Part **(C)** is used to study the angle between a line and a plane. Gestures supplement and emphasize the definition of the angle between a line and a plane. Part **(D)** is used to prove the perpendicular relationship between lines and planes. Gestures supplement and emphasize the method of proving the perpendicularity of planes by using a line to be perpendicular to two intersecting lines.

Based on high school mathematics textbooks and in combination with cognitive load theory and multimedia learning theory, researchers and front-line teachers jointly developed gesture and GeoGebra teaching courseware as well as teaching materials that integrate GeoGebra and gestures. Among them, the GeoGebra teaching courseware is based on the multimedia design principles in the multimedia learning theory (Mayer, 2014) to simulate the spatial relationships and complex abstract spatial graphics in three-dimensional geometry. The gestures of teachers mainly simulate spatial relationships and spatial figures in three-dimensional geometry, and they belong to descriptive gestures. Teaching materials that integrate GeoGebra with gestures complement each other’s advantages and deeply integrate the two rather than simply superimpose them. Some teaching materials are shown in Figures 2, 3.

### 3.5 Measuring tools

#### 3.5.1 Measuring tools for SA

The SA Test was implemented in the study using the Purdue Spatial Visualization Test (PSVT) designed by Bodner and Guay (1997). The test consists of three parts: developments, rotations and views Directions. The tests, respectively, correspond to students’ spatial visualization, mental rotation and spatial orientation abilities. There are a total of 36 test items, all of which are objective. One point will be awarded for each correct answer and 0 points for each incorrect one. The test is completed within 30 min. The test consists of a basic information module and a test item module. The basic information includes name, age, gender and the difficulty level of handling spatial content. The measurement tool demonstrated satisfactory reliability in this study, with Cronbach’s *α* = 0.710 and test–retest reliability = 0.788, supporting its use for assessing spatial ability as a key variable.

#### 3.5.2 Measurement of 3D-GT

The research divides 3D-GT into five levels based on Van Hiele’s theory, and then develops test items according to the five levels. The types of questions include single-choice questions, multiple-choice questions and subjective questions. Invite three front-line mathematics teaching experts to form a question item review panel. The matching degree between the review items and the five 3D-GT levels, as well as the matching degree between the test content and the students’ learning conditions. Ultimately, a 3D-GT level test consisting of 13 items was formed, including 7 single-choice questions, each worth 5 points, and 1 multiple-choice question.5 points will be awarded for selecting all correct options, 3 points for selecting only some correct options, and no points will be given for incorrect or multiple selections. There are 2 subjective questions. Each subjective question is divided into two sub-questions. The first sub-question is worth 7 points and the second sub-question is worth 8 points. The test items were predicted in another class of the same grade in the school, with 53 students participating in the test. The study conducted reliability and validity tests, one-dimensional tests, Item fit analysis, Wright Map analysis and Differential Item Functioning (DIF) for the test items based on the Rasch model using Winsteps Version 3.74.0 software. The important indicators for evaluating the measurement Reliability and validity of the Rasch model include Person and Item Separation and Person and Item reliability. The analysis results show that Person Separation = 2.28, Person Reliability = 0.84, Item Separation = 5.36, Item Reliability = 0.97. Linacre (2006) believed that if Person Separation >2, the test items could distinguish between those with excellent performance and those with poor performance. If Item Separation is greater than 3, the test items have good structural validity. The estimation of the difficulty of the items is stable enough to reliably sort the questions by difficulty. Person and Item Reliability >0.8 will be considered satisfactory (Linacre, 2012). This means that the test items meet the basic requirements and standards. One of the assumptions of the Rasch model is dimensionality. The criterion for dimensionality is that the variance explained by the measurement dimension should be at least 40% (Linacre, 2006). The variance explained by the first principal component of the residual shall not exceed 15% (McCreary et al., 2013). The minimum ratio between the variance explained by the measurement dimension and that explained by the first principal component of the residuals is selected as 3:1 (Embretson and Reise, 2000). The results show that Raw variance explained by measures = 49.7% > 40%. Eigenvalue = 12.8. Unexplned variance in first contrast = 8.7% < 15%, the ratio of the variance explained by the measurement dimension to that explained by the first principal component of the residual is approximately 5.7 > 3. This means that the test items satisfy the one-dimensional assumption. The research utilizes the fitting statistics to test the item function. The commonly used fitting metrics are INFIT and OUTFIT Mean Squares (MNSQ) (Boone et al., 2014). INFIT is more sensitive to abnormal data where the subject’s ability level is comparable to the difficulty of the question item, while OUTFIT is sensitive to the difference between the observed response and the expected response (Tennant and Conaghan, 2007). Linacre (2012) considered that the values of INFIT and OUTFIT MNSQ ranging from 0.5 to 1.5 were acceptable. The fitting analysis results showed that 0.54 < INFIT MNSQ <1.38, 0.49 < OUTFIT MNSQ < 1.80, among which ITEM T12 OUTFIT MNSQ = 0.49. ITEM T8 OUTFIT MNSQ = 1.53, ITEM T6 OUTFIT MNSQ = 1.80, and the OUTFIT MNSQ of other items are all between 0.56 and 1.30. Through the review of some predictions and student interviews, it was found that students with high abilities answered the multiple-choice questions T6 and T8 wrongly due to carelessness and misunderstanding of the question meaning, while students with very low abilities guessed the multiple-choice questions T6 and T8 correctly. This situation is a frequent occurrence in daily practical tests and is also acceptable. This means that the overall fitting of the test items is acceptable. Wright Map can place Person Ability and Item Difficulty on the same Logit Scale for direct comparison. The analysis results of the Wright Map can be found in Supplementary Figure 1. The distribution of Person Ability and Item Difficulty in Supplementary Figure 1 overlaps well, which means that the test items are applicable to the current sample level. DIF refers to the situation where different groups with the same ability level perform differently in the same project. Linacre (2012) holds that DIF CONTRAST > 0.5 is the dividing point for significant differences. The study conducted a DIF analysis on the initial test data based on gender grouping. The results showed 0.05 < |DIF| < 0.46. This means that there is no significant difference in the performance of male and female students in each item. Based on the above analysis results, the developed test items can better test students’ 3D-GT. After the test was conducted in three classes, two raters were invited to rate 30% of the data. The consistency of the raters was relatively high (Kappa = 0.89, *p* < 0.001), so one of them completed the remaining marking.

### 3.6 Data analysis

The study used SPSS 25 software to conduct descriptive analysis and inferential analysis on the test results. Inferential analysis mainly adopts one-way ANOVA, Analysis of covariance (ANCOVA), and two factors ANOVA. Independent-sample *T*-test, Paired sample *t*-test and Hierarchical Regression Analysis.

## 4 Results

### 4.1 Descriptive statistical analysis of the results

**Figure 4:**
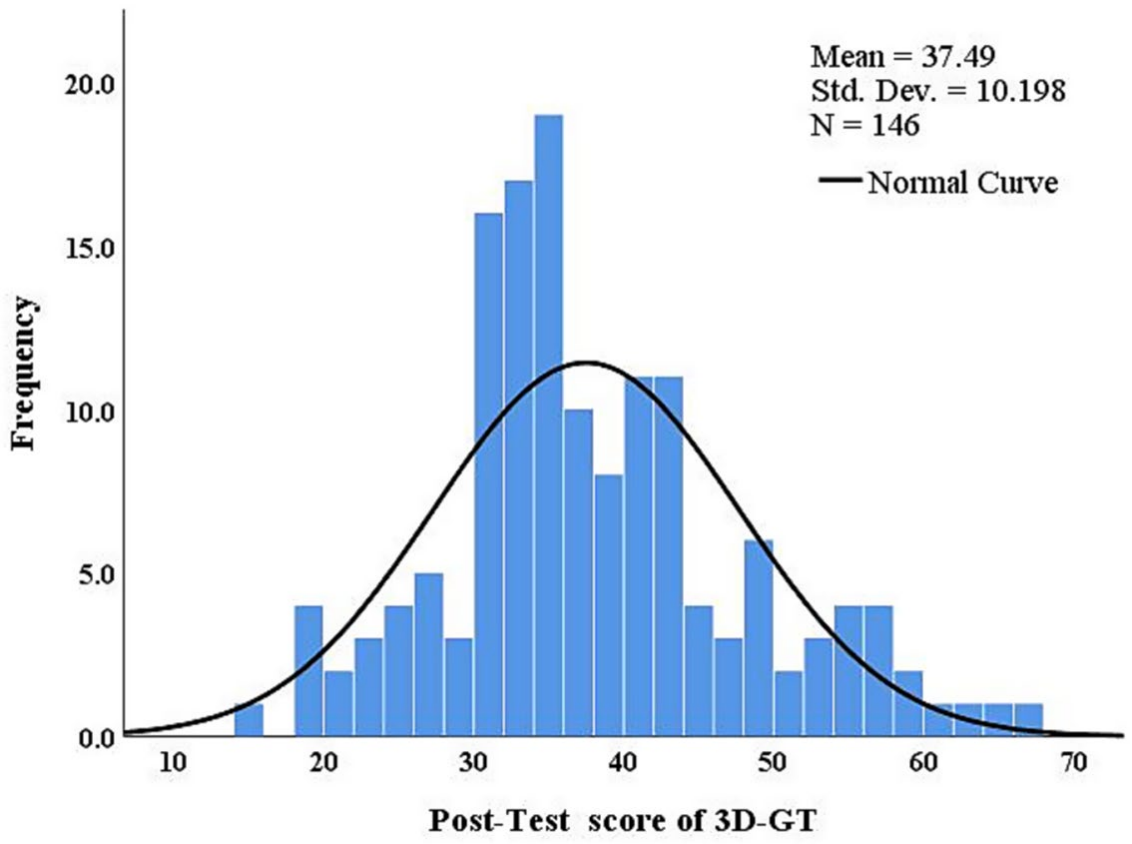
Histogram of post-test score of 3D-GT.

The study conducted descriptive statistical analysis, normality test and homogeneity of variance test on Pre-test scores of SA, Post-test scores of SA and Post-test score of 3D-GT. The results show that both Pre-test and post-test scores of SA satisfy the assumptions of normality and homogeneity of variance. The Post-test score of 3D-GT does not conform to the normality hypothesis, but conforms to the homogeneity of variance hypothesis (See Table 1). The study further analyzed the histogram of the Post-test score of 3D-GT (see Figure 4) and found that there was no significant deviation from the normal curve. Although the Kolmogorov–Smirnov normality test indicated that the data deviated from the normal distribution (*p* < 0.05). Based on the central limit theorem, sample size and combined with histogram analysis, we believe that the sample basically satisfies normality. The study conducted a one-way ANOVA on the Pre-test scores of SA of the three groups through SPSS 25 software, showing no significant difference. *F*(2,143) = 0.582, *p* = 0.56 > 0.05.

**Table 1:** Descriptive statistical analysis.

Test	Group	*N*	*M*	SD	Kolmogorov–Smirnov		Levene	
					Statistic	Sig	Statistic	Sig
Pre-test (SA)	Control	48	21.48	6.937				
	Experimental 1	49	22.39	5.737				
	Experimental 2	49	22.73	4.907				
	Total	146	22.21	5.894	0.069	0.087	2.223	0.112
Post-test (SA)	Control	48	21.90	6.873				
	Experimental 1	49	25.55	6.371				
	Experimental 2	49	25.24	6.217				
	Total	146	24.25	6.655	0.070	0.080	0.082	0.922
Post-test (3D-GT)	Control	48	33.33	9.318				
	Experimental 1	49	39.39	9.434				
	Experimental 2	49	39.67	10.693				
	Total	146	37.49	10.198	0.103	0.001	1.376	0.256

### 4.2 Differences in the impact of different teaching methods on SA

**Figure 5:**
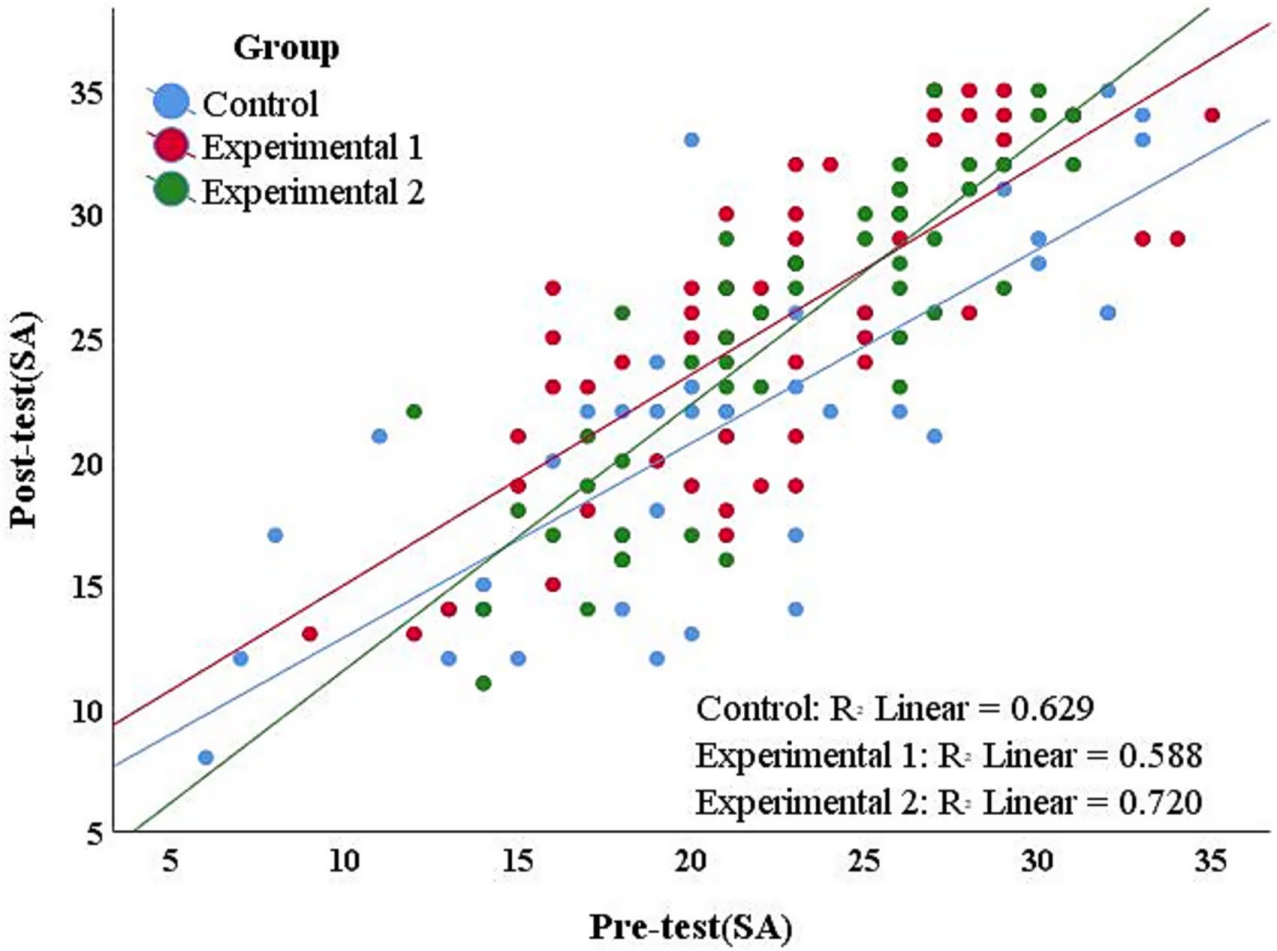
Scatter plot of pre-test and post-test scores of SA.

To enhance the statistical testing power and control any minor variations at the baseline level. This study first selected ANCOVA with Pre-test scores of SA as the covariate to examine the influence of different teaching methods on Post-test scores of SA. The study plotted scatter plots using SPSS 25 software to show the linear positive correlation between covariates and dependent variables in each group (see Figure 5). The slope homogeneity test indicated that the interaction of Group * Pre-test of SA was not significant, *F*(2,140) = 2.122, *p* = 0.124 > 0.05, satisfying the core hypothesis. The results of Levene’s homogeneity of variance test based on residuals were not significant, with *F* = 2.906 and *p* = 0.058 > 0.05. The Shapiro–Wilk test indicated that the residuals were normally distributed, *p* = 0.186 > 0.05. This means that all the presuppositions of ANCOVA hold true, and the ANCOVA results show (see Table 2). After controlling for the influence of Pre-test of SA, different teaching methods had no significant main effect on students’ Post-test of SA and the effect size was small, *F*(2,140) = 1.370, *p* = 0.258 > 0.001, *η*^2^ = 0.019. The effect of students’ Pre-test score of SA on Post-test score of SA was significant, *F*(1,140) = 247.405, *p* = 0.000 < 0.001, *η*^2^ = 0.639. Further Correlation analysis results of students’ Pre-test scores of SA and Post-test scores of SA showed that the Pearson Correlation coefficient was as high as 0.788, *p* < 0.001.

**Table 2:** The results of the covariance analysis of the post-test of SA.

Source	Type III sum of squares	df	Mean square	*F*	Sig	Partial eta squared
Corrected Model	4,275.519^a^	5	855.104	55.795	0.000	0.666
Intercept	146.976	1	146.976	9.590	0.002	0.064
Group	41.989	2	20.994	1.370	0.258	0.019
Pre-Test SA	3,791.670	1	3,791.670	247.405	0.000	0.639
Group * Pre-Test SA	65.058	2	32.529	2.122	0.124	0.029
Error	2,145.604	140	15.326			
Total	92,254.000	146				
Corrected Total	6,421.123	145				

a *R* Squared = 0.666 (Adjusted *R* Squared = 0.654).

### 4.3 The differences in the impact of different teaching methods on 3D-GT

**Figure 6:**
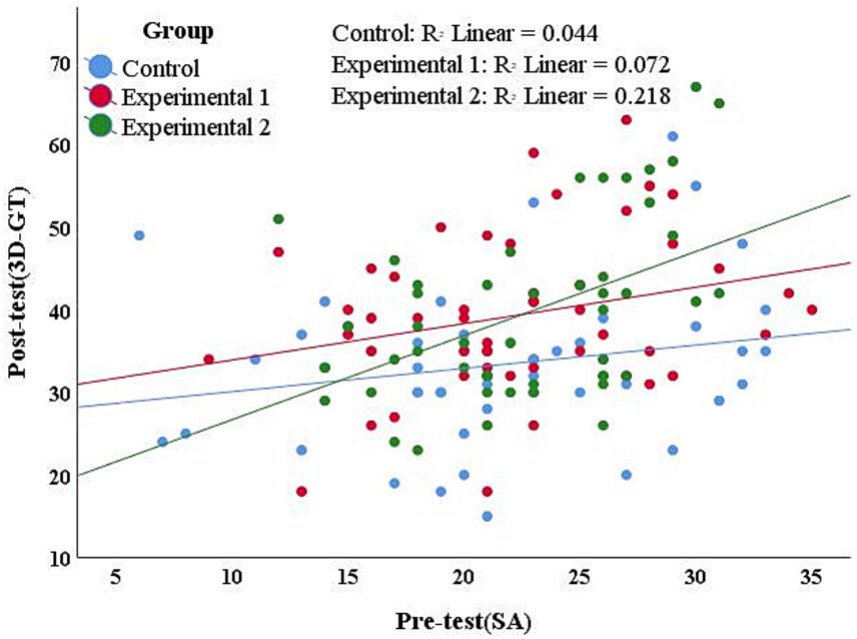
Scatter plot of SA pre-test scores and 3D-GT post-test scores.

To enhance the statistical test power and control any minor variations at the baseline level. The study first selected ANCOVA with Pre-test scores of SA as a covariate to examine the influence of different teaching methods on 3D-GT. The scatter plot was plotted using SPSS 25 software to show that the linear correlation between covariates and dependent variables was extremely low within each group (see Figure 6). This means that it does not conform to the basic assumptions of ANCOVA. Therefore, in this study, a two-factor ANOVA was conducted to examine the differential effects of various teaching methods on the 3D-GT performance of students with high and low SA groups. Students were divided into high and low SA groups based on the median of their pre-test SA scores. The calculation using SPSS 25 indicated that the median score was 22 (*n* = 5). To maintain the clarity of group definitions and ensure valid comparisons, students with scores greater than the median were classified into the high SA group, while those with scores less than or equal to the median were assigned to the low SA group. An independent samples *T*-test confirmed a significant difference between the high and low SA groups, *t*(144) = −16.603, *p* < 0.05. The descriptive statistics related to the high and low SA groups are shown in Table 3.

**Table 3:** Pre-test grouping situation of SA.

Pre-test SA	Group	Post-test (3D-GT)				
		N	M	SD	Levene statistic	Sig.
High-ability	Control	22	36.50	9.927	1.585	0.168
	Experimental 1	24	42.38	9.659		
	Experimental 2	25	44.04	11.735		
	Total	71	41.14	10.850		
Low-ability	Control	26	30.65	8.010		
	Experimental 1	25	36.52	8.432		
	Experimental 2	24	35.13	7.255		
	Total	75	34.04	8.221		

**Figure 7:**
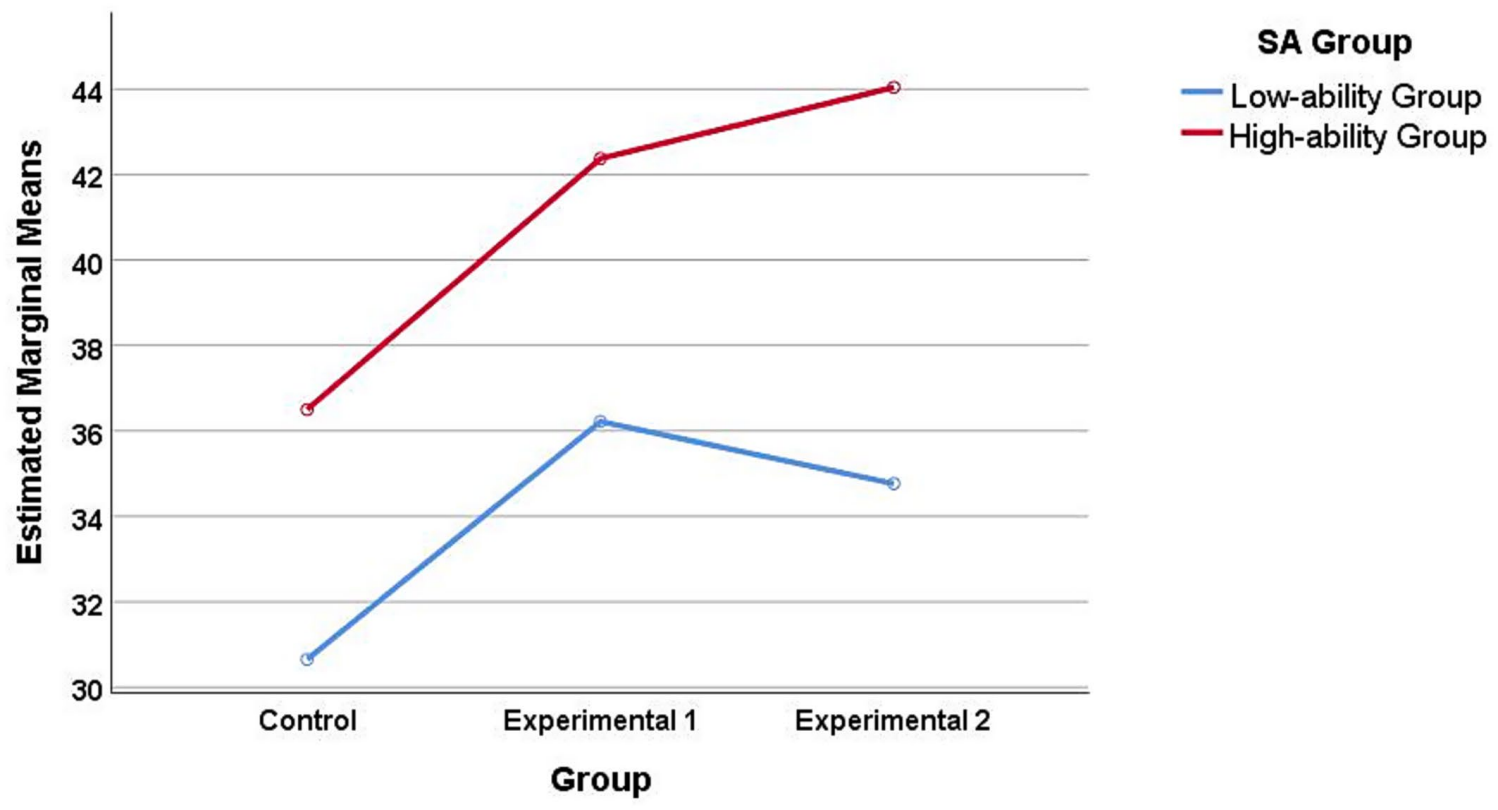
The estimated marginal mean of the post-test scores of 3D-GT.

The Two-factor ANOVA was conducted using SPSS 25, and the estimated marginal mean plots were plotted (see Figure 7). The post-test scores of students in the high SA group showed a gradually increasing trend under the three teaching methods. In contrast, the students in the low SA group performed the best in the gesture teaching method, but showed a decline in the teaching method that integrates GeoGebra and gestures. Although the above descriptive model deserves attention, the interaction between the teaching method and SA Group is not significant, *F*(2, 140) = 0.444, *p* = 0.643, *η*^2^ = 0.006. The main effect of the teaching method was significant, *F*(2, 140) = 6.060, *p* = 0.002 < 0.05, *η*^2^ = 0.086. The main effect of SA Group was also significant, *F*(1, 140) = 19.987, *p* = 0.000 < 0.001, *η*^2^ = 0.125. For detailed results, please refer to Table 4.

**Table 4:** Two-factor ANOVA for pre-test of SA and teaching methods.

Source	Type III sum of squares	df	Mean square	*F*	Sig	Partial eta squared
Corrected model	3,039.659^a^	5	607.932	7.068	0.000	0.202
Intercept	205,142.148	1	205,142.148	2,385.208	0.000	0.945
Group	1,131.057	2	565.529	6.575	0.002	0.086
SA group	1,719.016	1	1,719.016	19.987	0.000	0.125
Group * SA group	76.311	2	38.155	0.444	0.643	0.006
Error	220,318.000	140	86.006			
Total	15,080.493	145				

a *R* Squared = 0.202 (Adjusted *R* Squared = 0.173).

To ensure robustness and avoid information loss due to dichotomization, the study employed hierarchical regression analysis as a robustness check. The pre-test SA was regarded as a continuous moderating variable, and the interaction between teaching method and SA was modeled (Aiken and West, 1991; Hayes, 2022). The result pattern was highly consistent with the results of variance analysis, providing convergent evidence. Block 1 included different teaching groups and pre-test SA to predict the 3D-GT post-test, explaining 16.6% of the variance (*R*^2^ = 0.166, *F*(3,142) = 9.410, *p* < 0.001). Block 2 added interaction terms, which slightly increased the explained variance (Δ*R*^2^ = 0.028, F_change(2,140) = 2.440, *p* = 0.091 > 0.05).

Due to the insignificant interaction, the main effects of the teaching methods and SA groups were significant. Therefore, the study directly conducts a *post hoc* comparative analysis of teaching methods and SA, respectively. A post hoc comparative analysis of the 3D-GL scores of different teaching methods revealed. There was a significant difference between the Control group and the Experimental group 1, *p* = 0.002 < 0.05. There was also a significant difference between the Control group and the Experimental group 2, *p* = 0.001 < 0.05. There was no significant difference between Experimental Group 1 and Experimental Group 2, *p* = 0.879 > 0.05. The independent samples *t*-test indicated that the 3D-GL scores of the high and low SA groups were significantly different, *t*(144) = −4.472, *p* < 0.05.

## 5 Discussion

Given that both the single dynamic visualization technology and the gesture teaching method have limitations in their impact on SA and 3D-GT. Few studies have focused on the differences in the impact of various teaching methods in three-dimensional geometry on students’ SA and 3D-GT. In order to promote personalized teaching of three-dimensional geometry content and achieve the adaptation of teaching methods to students’ initial SA characteristics. Based on the theory of multimedia learning and cognitive load, this study designed a teaching method that integrates GeoGebra and gestures. The differences in the independent effect and the integrated effect between the teaching method supported by GeoGebra and the gesture teaching method were systematically explored, as well as the moderating effect of students’ initial SA therein. The research results show that the main effect of teaching methods on the post-test of students’ SA is not significant. The interaction between students’ initial SA and teaching methods is not significant, while the main effect of students’ initial SA is significant. In addition, there are significant differences in the influence of different teaching methods on students’ 3D-GT level. The main effects of teaching methods and pre-test SA are both significant, but the interaction is not significant. Although baseline equivalence across groups was tested and supported, the inherent limitations of the quasi-experimental design cannot be eliminated. Potential selection effects and unmeasured classroom-level variables therefore cannot be fully ruled out. Accordingly, the following discussion focuses on interpreting associations rather than inferring causal mechanisms.

### 5.1 There is no significant difference in the effect of different teaching methods on students’ post-test SA scores

The ANCOVA results showed that after controlling for the influence of Pre-test of SA, the main effect of the teaching method on students’ Post-test of SA was not significant and the effect size was small. However, there were significant differences between the front-side and back-test SA scores of different SA groups, and all showed an increasing trend. Among them, for the high SA group, *t*(70) = −4.585, *p* < 0.001; for the low SA group, *t*(71) = −3.560, *p* < 0.001. This means that there is no significant difference among different teaching groups, but students with high and low SA have all improved to a certain extent. This finding is consistent with the results of existing meta-analysis studies indicating that spatial capabilities have a certain degree of plasticity (Uttal et al., 2013). However, there are multiple possible reasons for the improvement of spatial capabilities. From the perspective of cognitive mechanisms, this result might be due to the fact that the core of the teaching methods in the research are all visual and operational teaching strategies, and cognitive mechanisms are all the ability to create and operate mental representations in the mind. Because such similar teaching strategies and cognitive mechanisms may be the real factors that promote the improvement of students’ SA, rather than the medium itself (Clark, 1994). Therefore, there is no significant difference in the effects produced by different teaching methods. From the perspective of teaching content, this result may also be due to the influence of three-dimensional geometry content learning on students’ SA (Wai et al., 2009). Because the interval between the test results before and after is 11 weeks, the possibility of the influence from the practice effect is relatively small. The research results show that the three different teaching methods have no significant difference in the post-test effect on SA and do not produce a synergistic gain effect. This thus rejects Hypothesis 1.

### 5.2 The initial SA of the students did not significantly moderate the effect of the teaching method on the post-test SA

The ANCOVA results show that the main effect of students’ initial SA on the post-test scores of SA is significant, and the interaction with teaching methods is not significant. The results of the Correlation analysis showed that the Pearson Correlation coefficient between students’ initial SA and their post-test scores of SA was as high as 0.788, with *p* < 0.001. This finding is consistent with the theoretical consensus in psychometrics that SA is regarded as a relatively stable cognitive trait (Lohman, 1996). Although meta-analysis indicates that spatial capabilities are plastic (Uttal et al., 2013). Experimental teaching intervention may have produced a certain value-added effect on the basis of stabilizing traits, but it has not completely changed the relative influence effect of the pre-test SA. This means that students’ initial SA does not significantly moderate the impact of different teaching methods on the post-test of SA, and the pre-test score of students’ SA is the main predictor of the post-test score. This thus rejects Hypothesis 3.

### 5.3 The different teaching methods had significant differences in influencing the post-test scores of students’ 3D-GT

The research results show that compared with the pure GeoGebra teaching method, pure gesture teaching and the teaching method integrating GeoGebra and gestures have a significant impact on students’ 3D-GT. However, there is no significant difference between the pure gesture teaching method and the teaching method integrating GeoGebra and gestures on students’ 3D-GT. Therefore, Hypothesis 2 is established. However, this result does not indicate that the intervention of gestures is necessarily more effective than that of GeoGebra. This result can be explained by cognitive load theory. The dynamic visualization presented by the GeoGebra teaching method is extremely susceptible to individual differences (Hegarty and Waller, 2004; Huk, 2006), and it is difficult to flexibly represent its local spatial relationships. This does not benefit cognitive load management in students with low SA. Because the gesture teaching method does not place an excessive burden on working memory resources (Paas and Sweller, 2012), it can save students’ cognitive resources (Ping and Goldin-Meadow, 2010). Students with different initial SA had lower cognitive load. The teaching method integrating GeoGebra and gestures effectively reduces the cognitive load of students with different initial SA. Gestures in integrated teaching methods can promote students’ spatial cognition (Alibali, 2005) by describing local three-dimensional spatial relationships (Atit et al., 2015) and reduce students’ internal load. In the integrated teaching method, GeoGebra uses dynamic visualization to demonstrate intuitive graphics, which is helpful to reduce students’ external cognitive load.

### 5.4 The influence trend of GeoGebra and gesture integrated teaching methods on the 3D-GT of students with different initial SA

Although the results of the two-factor ANOVA and hierarchical regression analysis indicated that there was no significant interaction between teaching methods and SA, the estimated marginal mean plot revealed certain descriptive trends under different teaching conditions. Specifically, for students with higher initial SA, the teaching method that integrates GeoGebra and gestures resulted in slightly higher 3D-GT scores compared to the pure GeoGebra or pure gesture conditions. Conversely, for students with lower initial SA, the performance under the integrated GeoGebra and gesture teaching method tended to be lower than that of the gesture-only condition. It should be emphasized that these differences did not reach statistical significance, so they should be interpreted as descriptive patterns rather than confirmatory evidence. From a theoretical perspective, these descriptive trends may be tentatively interpreted in light of cognitive load theory. The high SA group has lower cognitive load, which can not only benefit from the dynamic visualization provided by GeoGebra (Huk, 2006), but also promote learning in non-corresponding gesture descriptions (Brucker et al., 2022). Therefore, compared with pure gesture teaching and GeoGebra teaching, the high SA group may gain greater benefits from the teaching method integrating GeoGebra and gestures. The intrinsic cognitive load of the low SA group is inherently high during the three-dimensional geometry learning process. Under the teaching method that integrates GeoGebra and gestures, the low SA group is prone to increase external cognitive load due to the dynamic visualization and fusion method provided by GeoGebra (Huk, 2006). Gesture teaching not only helps save cognitive resources (Ping and Goldin-Meadow, 2010), but also interacts with spatial thinking (Brooks et al., 2018).

### 5.5 Students’ initial SA did not significantly moderate the impact of teaching methods on 3D-GT scores

The unexpected result is that the interaction between SA group and teaching method is not significant, thus Hypothesis 4 is rejected. There are several possible reasons for this result. From the perspective of cognitive load theory, different teaching methods can help reduce students’ cognitive load, and students have more working memory resources to solve more complex problems (Sweller et al., 2019), which has a general positive effect on students’ 3D-GT. Interpreted from the perspective of multimedia learning theory, this result may be because different teaching methods promote students’ cognitive processing through different encoding methods (Mayer, 2014), which has a generally positive effect on students’ 3D-GT. The significant main effect of teaching method also implies that the teaching method in the research process was successful and fair. From the perspective of individual differences, although the teaching method contributes to the development of 3D-GT, it cannot make up for the impact of differences in students’ SA (Cronbach and Snow, 1977). In the research results, the SA group effect is significant and greater than the effect of teaching method, which is an important manifestation of the main effect of ability.

### 5.6 The overall impact of students’ initial SA on 3D-GT is greater than the impact of teaching methods

The research results show that the main effect of SA group is significant and greater than the main effect of teaching method. This result is consistent with findings from previous studies (Izzati, 2024; Heo and Toomey, 2020), that is, SA has the most significant overall impact on learning outcomes. It also further verified the close relationship between SA and 3D-GT (Pittalis and Christou, 2010). This result can be explained by the theory of meaningful learning, where learners already know what is the single most important factor affecting learning (Ausubel, 1968). SA is an important part of human ability (Geary, 2022), and it is also the main factor affecting 3D-GT.

## 6 Conclusions and implications

Given that both the single dynamic visualization technology and the gesture teaching method have limitations in their impact on SA and 3D-GT. Few studies have focused on the differences in the impact of various teaching methods in three-dimensional geometry on students’ SA and 3D-GT. In order to promote personalized teaching of three-dimensional geometry content and achieve the adaptation of teaching methods to students’ initial SA characteristics. Based on the theory of multimedia learning and cognitive load, this study designed a teaching method that integrates GeoGebra and gestures. The differences in the independent effect and the integrated effect between the teaching method supported by GeoGebra and the gesture teaching method were systematically explored, as well as the moderating effect of students’ initial SA therein. The study has the following important findings: (1) The three teaching methods have no significant impact on students’ SA, but the pre- and post-test SA scores are significantly improved. (2) Compared with GeoGebra teaching, the teaching method of gestures and the integration of GeoGebra and gestures has a significant impact on students’ 3D-GT. Compared with gesture teaching, the teaching method of integrating GeoGebra and gestures has no significant impact on students’ 3D-GT. (3) Students’ initial SA did not significantly moderate the impact of teaching methods on the development of SA and 3D-GT, and was the main predictor of post-test scores on SA and 3D-GT. (4) Compared with pure GeoGebra and gesture teaching methods, the 3D-GT of the high SA group in the teaching method integrating GeoGebra and gestures showed an upward trend. Compared with the pure gesture teaching method, the 3D-GT of the low SA group showed a downward trend in the teaching method integrating GeoGebra and gestures. It should be emphasized that these differences did not reach statistical significance. (5) Students’ initial SA has a greater overall impact on 3D-GT than teaching methods.

The results confirm that SA is both malleable and stable and is an important predictor of 3D-GT. This enlightens teachers to focus on cultivating students’ SA in a long-term and targeted manner before teaching three-dimensional geometry. Short-term training and training are difficult to reduce the differences between students with different initial SA. Teachers’ long-term focus on cultivating students’ SA before teaching three-dimensional geometry is more conducive to promoting the development of students’ 3D-GT than choosing three-dimensional geometry teaching methods that match their academic conditions. In terms of cultivating 3D-GT, this study suggests that teachers use gesture teaching methods or teaching methods that integrate GeoGebra and gestures to be more conducive to students’ development of 3D-GT. The research results show that students with different initial SA have different trends in 3D-GT performance under different teaching methods. Although the trend differences are not significant, it can provide an exploratory path for teachers to select solid geometry teaching methods that match the academic performance of students. The teaching intervention design that integrates GeoGebra and gestures proposed in the study provides a reference for other researchers and front-line teachers in the design of SA training programs. However, it should be noted that the integration of teaching tools should focus on effectiveness and avoid simple superposition, which does not necessarily produce a gain effect.

## 7 Limitations and future research

This study still has certain limitations, and future research can be further deepened on this basis. First of all, Due to the lack of random assignment, although we were able to demonstrate different outcomes associated with teaching methods, we were unable to rule out all other explanations for these differences. Future research should aim to draw more reliable causal conclusions through randomized controlled trials. Secondly, SA is a multi-dimensional construct. This study mainly selected three dimensions for discussion based on the three-dimensional geometry content in mathematics textbooks. However, there may also be influences from other dimensions of SA during three-dimensional geometry learning. This study failed to fully examine the impact of these underlying dimensions. Thirdly, different teaching methods may have different effects on each dimension of 3D-GT. This study has not yet conducted a detailed analysis of the sub-dimensions of 3D-GT. Further exploration of such differences will help provide more targeted and operational reference for front-line teaching. Fourthly, the median split approach, while convenient for group comparisons, may reduce statistical power and obscure individual variability by dichotomizing a continuous construct. Future studies should adopt analytic strategies that retain the continuous nature of spatial ability to enhance precision and robustness.

## Data Availability

The raw data supporting the conclusions of this article will be made available by the authors, without undue reservation.
