# Supplementary material for: Research on the impact of different teaching methods on students’ spatial ability and three-dimensional geometric thinking
Source: Front Psychol. 2026 Feb 12;17:1744734. doi: 10.3389/fpsyg.2026.1744734 (PMC12935921; doi:10.3389/fpsyg.2026.1744734)
Supplement: Supplementary file 1 [file Supplementary_file_1.docx]

Supplementary Material

# Supplementary Figures


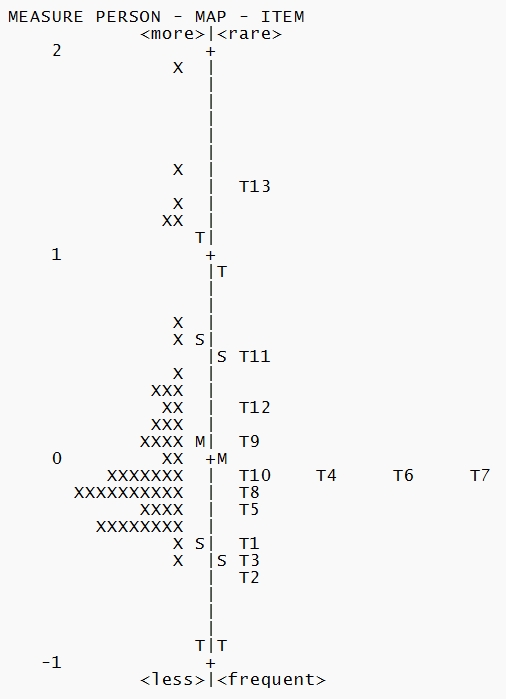


**Supplementary Figure 1.** The Wright person-item map
